# Effects of chronic taurine administration on healthy pregnant rats and the consequences on the offspring: Datasets for motor tests and oxidative stress

**DOI:** 10.1016/j.dib.2022.108015

**Published:** 2022-03-03

**Authors:** Viridiana Vargas-Castro, Ricardo Gomez-Diaz, Victor M. Blanco-Alvarez, Constantino Tomas-Sanchez, Alejandro Gonzalez-Vazquez, Ana Karina Aguilar-Peralta, Juan A. Gonzalez-Barrios, Daniel Martinez-Fong, Jose R. Eguibar, Araceli Ugarte, Guadalupe Soto-Rodriguez, Eduardo Brambila, Lourdes Millán-Perez Peña, Bertha Alicia Leon-Chavez

**Affiliations:** a Facultad de Ciencias Químicas, benmérita Universidad Autónoma de Puebla, Puebla, C.P. 72570. México; b Facultad de Enfermería, Benemérita Universidad Autónoma de Puebla, Puebla, C. P. 72304, México; c Laboratorio de Medicina Genómica, Hospital Regional 1º de Octubre, ISSSTE, Mexico City C. P. 07760, Mexico; d Departamento de Fisiología, Biofísica y Neurociencias, Centro de Investigación y de Estudios Avanzados del Instituto Politécnico Nacional, Apartado Postal 14-740, Mexico City, C. P. 07000, Mexico; e Nanoparticle Therapy Institute 404 Avenida Monte Blanco Aguascalientes, Aguascalientes, C.P. 20120, México.; f Instituto de Fisiología, Benemérita Universidad Autónoma de Puebla, Puebla, PU C. P. 72570, México; g Facultad de Medicina, Benemérita Universidad Autónoma de Puebla, Puebla, PU C. P. 72304, México

**Keywords:** Nitric oxide, Lipid peroxidation, Embryonic development, Sprague-Dawley, Vestibular imbalances, Muscle strength deficit

## Abstract

We present the data for taurine (2-aminoethanesulfonic acid) treatment to healthy pregnant Sprague Dawley rats (SD). At embryonic day 15 (E15), healthy pregnant SD rats were given taurine treatment (50 mg/L drinking water) and then to their male offspring until they reached the age of eight months. We quantify, in the offspring, the concentration of nitric oxide (NO) through the Griess colorimetric reaction [1] and malondialdehyde + 4-hydroxyalkenals (MDA + 4-HDA) by the Gérard-Monnier technique [2]. The assessment ages for NO and MDA + 4-HDA were at postnatal day 15 (PND15), 1, 3, and 8 months of age.

The body weight was measured along with the integral motor behavior in the perinatal stage through the surface righting reflex test at PND5, cliff aversion test at PND9, grip strength test at PND 11, and front limb and hindlimb suspension tests at PND13. The tests were performed accordingly with [3].

The data obtained showed that SD rats with the taurine administration performed poorly in the motor tests compared with the untreated healthy rats. The taurine-treated rats also showed increased lipid peroxidation preferentially in cerebral regions involved in motor activity, such as the medulla oblongata, the subcortical nuclei, and the cerebral cortex. However, the taurine treatment only increased NO concentration in the evaluated cerebral regions at older ages.

At E15, taurine plays a pivotal role in the excitatory/inhibitory neuromodulation, presumably by acting as an excitatory neurotransmitter during the GABA-switch [4]. The increase in the taurine concentration during the embryonic period might cause excitotoxicity in healthy brains, which might lead to impairments in the motor development of the offspring. Therefore, the present datasets can be valuable for researchers who attempt to use the taurine supplement on healthy animal models at gestational stages; and explore the relation with taurine intake during pregnancy in human patients. These datasets are related to the article “Long-term taurine administration improves motor skills in a tubulinopathy rat model by decreasing oxidative stress and promoting myelination” [5].

## Specifications Table

**Table:** 

Subject	Biological Sciences – Neuroscience Developmental
Specific subject area	Colorimetry and UV/Vis Spectroscopy, Behavioral analysis
Type of data	Graphs
How the data were acquired	Colorimetric Griess Reaction for nitrite detection Gérard-Monnier colorimetric reaction for MDA + 4-HDA quantification Set of Motor Tests SmartSpec 3000 spectrophotometer (Bio-Rad; Hercules, CA, USA)
Data format	Raw Analyzed
Description of data collection	The rats were divided into two groups: (1) male Sprague Dawley rats that received no therapy (SD), and (2) male Sprague Dawley rats that received taurine supplementation (SD/Taurine). To quantify nitrites and MDA+4-HDA in the supernatants, at least three brains from each group were obtained and manually homogenized. Motor and behavioral tests were carried out [3] in a minimum of 7 pups per group.
Data source location	Institution: Benemérita Universidad Autónoma de Puebla City/Town/Region: Puebla, Puebla Country: Mexico
Data accessibility	Repository name: Mendeley Data Data identification number: doi: 10.17632/b3srhpsr9y.1 Direct URL to data: https://data.mendeley.com/datasets/b3srhpsr9y/1
Related research article	V. Vargas-Castro, R. Gomez-Diaz, V.M. Blanco-Alvarez, C. Tomas-Sanchez, A. Gonzalez-Vazquez, A.K. Aguilar-Peralta, J.A. Gonzalez-Barrios, D. Martinez-Fong, J.R. Eguibar, C. Vivar, A. Ugarte, G. Soto-Rodriguez, E. Brambila, L. Millán-Perez-Peña, B.A. Leon-Chavez. Long-term taurine administration improves motor skills in a tubulinopathy rat model by decreasing oxidative stress and promoting myelination, Mol. Cell. Neurosci. 115 (2021) 103,643. https://doi.org/10.1016/j.mcn.2021.103643

## Value of the Data

• The relevance of the presented data is to provide information about taurine supplementation effects during pregnancy in healthy individuals, the effects on motor behavior, and the nitrosative/oxidative stress state of the offspring.
• These datasets might be helpful to research the effect of some energetic drinks during pregnancy and breastfeeding, as well as the possible consequences on motor development in newborns.
• These data might be used or reused to analyze the long-life effect of early exposure to taurine intake in young and adult subjects and the mechanisms (immunological, excitotoxic, etcetera) involved in the neurological damage.

## 1 Data Description

Raw Data from every evaluation has been provided as supplementary data files.

## 2 Experimental Design, Materials and Methods

### 2.1 Experimental design

CINVESTAV's vivarium supplied 3 months old, healthy-pregnant Sprague Dawley rats (MGI Cat#5,651,135, RRID: MGI:5,651,135). All pregnant rats were housed individually in acrylic cages (34 cm × 44 cm × 20 cm) in a suitable room with regulated temperature (an average of 22 ± 2 °C) and light-dark cycles (12:12 h; light starting at 07:00 h) until delivery. Once born, the litter remained in the same cage with the mother until one month old.

**Fig. 1:**
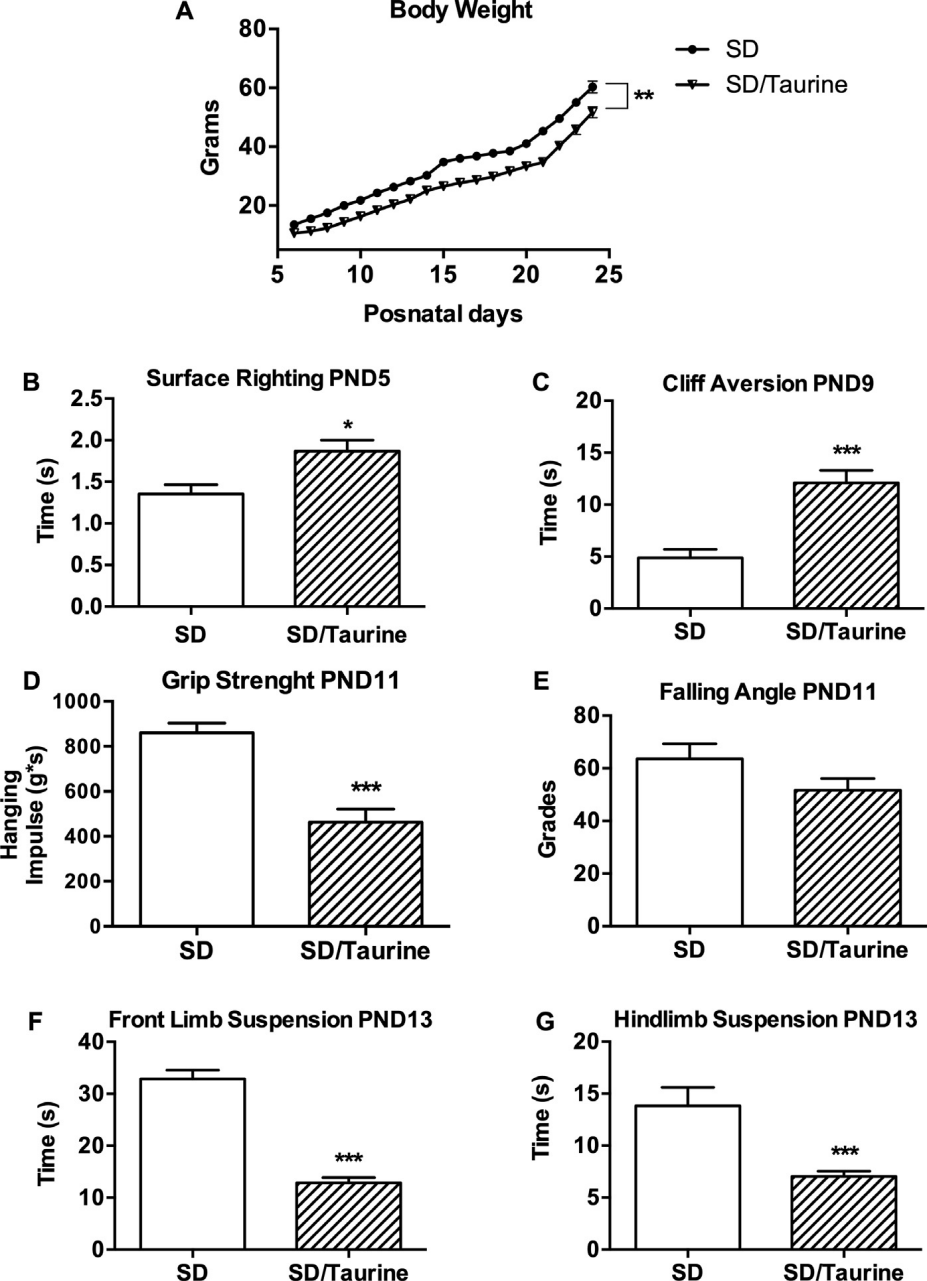
Neurotoxic effect of taurine administration on the body weight and motor behavior performance of healthy Sprague Dawley rats. (A) Significant reduction (from 14% to 29%) of the bodyweight of SD/Taurine compared with SD rats. (B) Increased time in the surface righting reflex of SD/Taurine by 48.0 ± 24.8% at PND5. (C) Increased time to avoid the cliff of SD/Taurine by 38.27 ± 9.8% at PND9 compared with SD rats. (D) Decreased grip strength of four limbs of SD/Taurine by 46.3 ± 3.7% at PND 11. (E) Lack of effect in the falling angle at PND 11. (F) Decreased suspension time of front limbs of SD/Taurine by 60.8 ± 3.0% at PND13. (G) Decreased hindlimb suspension time of SD/Taurine by 49.1 ± 3.7% at PND13. *P < 0.05, ** *P* < 0.01, ***P < 0.001 when SD/Taurine group vs. SD controls are compared using student's *t*-test.

**Fig. 2:**
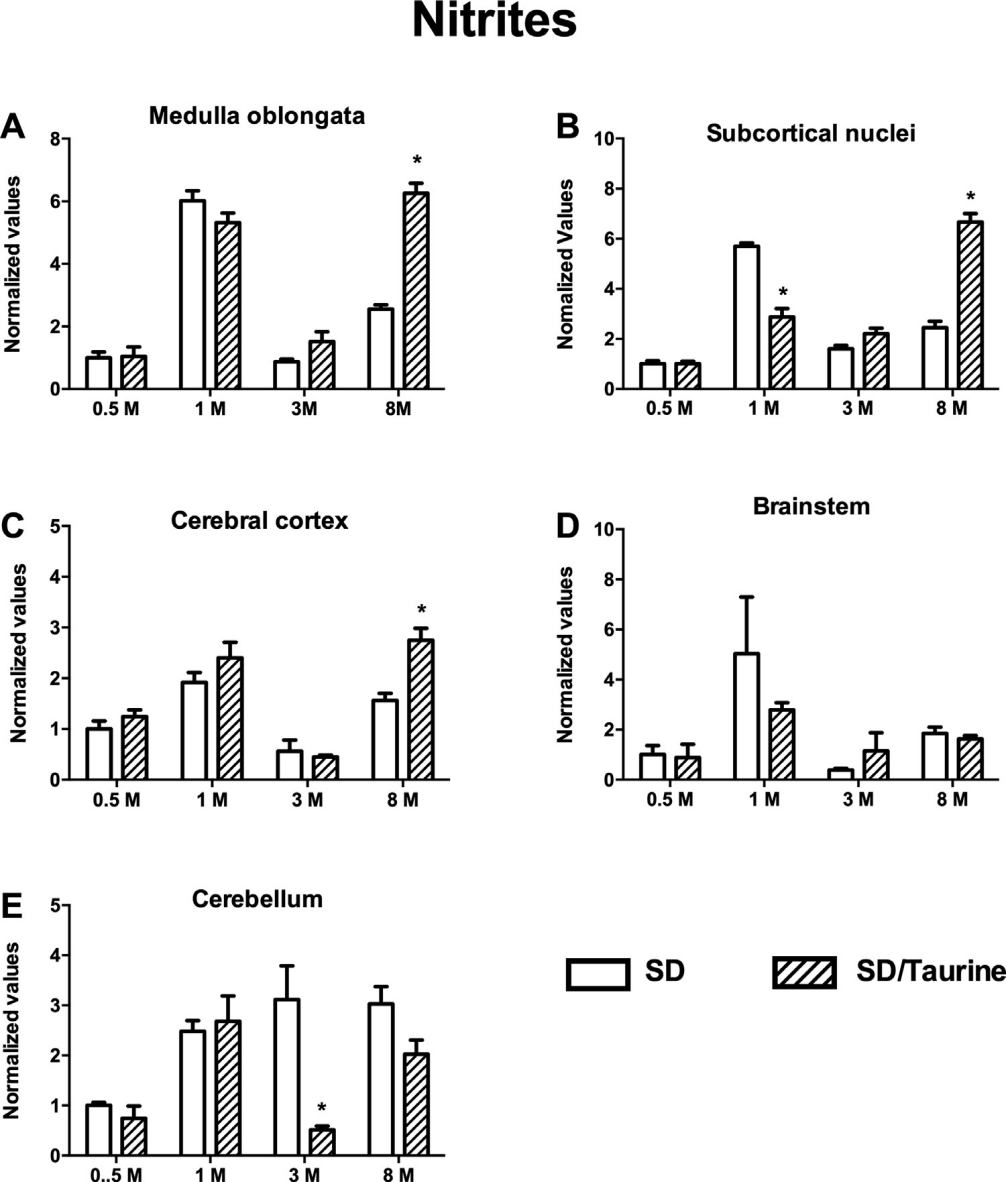
Taurine administration increased nitrosative stress only in some regions and ages evaluated in taurine-treated rats. (A) The increase in the medulla oblongata of the SD/Taurine group was 144.4 ± 12.4% at eight-month-old compared with the SD control. (B) The increase in subcortical nuclei of the SD/Taurine group was 171.9 ± 13.5% at 8-month-old. A significant decrease by 50 ± 6% occurred at one-month-old. (C) The increase in the cerebral cortex of the SD/Taurine group was 76.1 ± 15.2% at 8 months old. (D) Lack of effect in the brainstem. (E) Only a reduction by 83.44 ± 2.4% in the cerebellum of the SD/Taurine group at 3-month-old. The legends are common for all graphs. *P < 0.05 using multiple student's t-tests to compare the SD/Taurine group vs. SD controls.

**Fig. 3:**
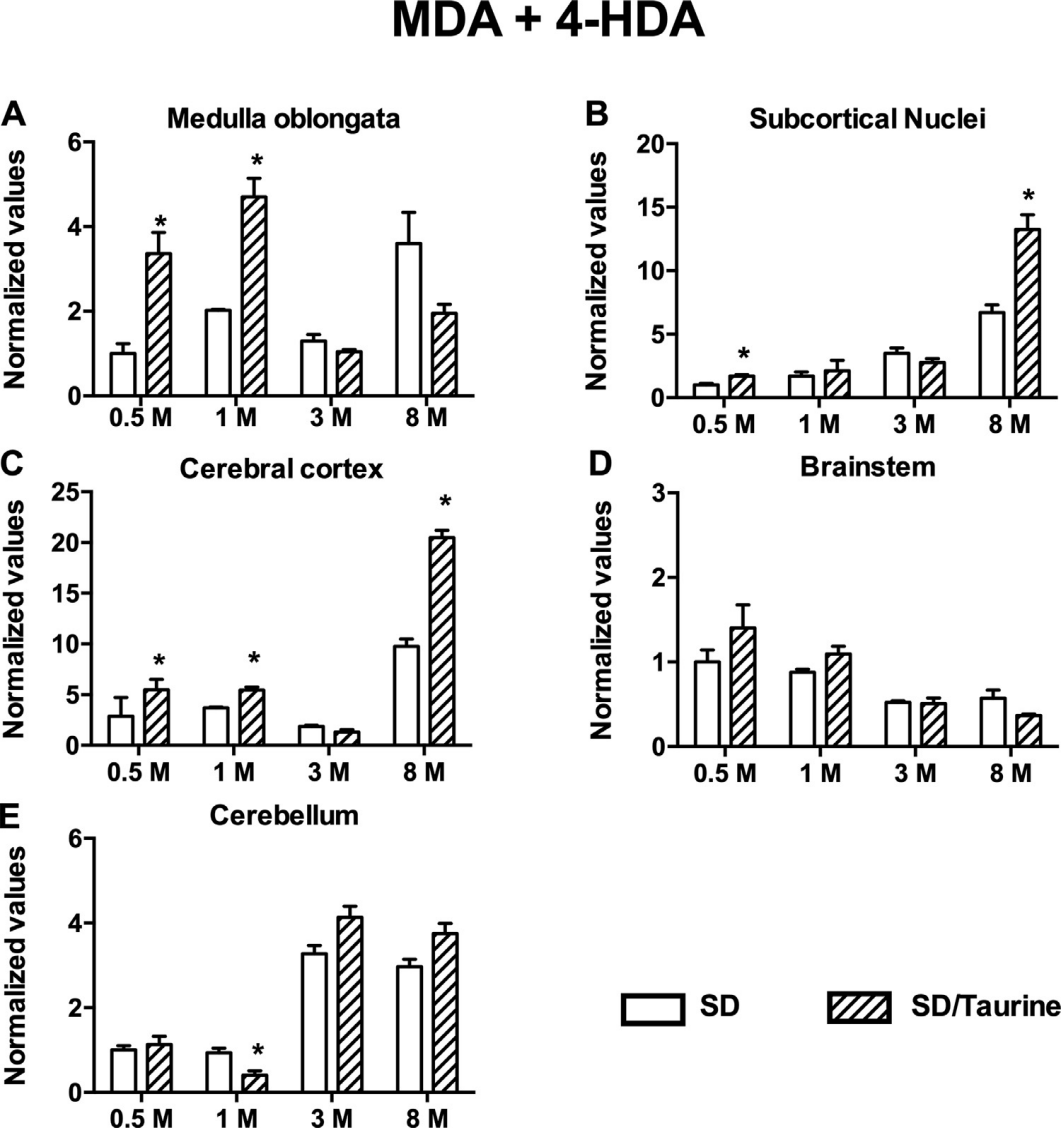
Taurine administration increased lipid peroxidation in brain regions of healthy Sprague Dawley rats. (A) The increase in medulla oblongata was 236.2 ± 50.4% at PND15 and 130.4 ± 21.7% at 1-month-old in the SD/Taurine group. (B) The increase in the subcortical nuclei was 97.8 ± 17.3% at 8-month-old. (C) The increase in the cerebral cortex was 53.8 ± 8.9% at one-month-old and 10 ± 7.22% at 8 months old in the SD/Taurine group. (D) No effect in the brainstem. (E) Only a reduction by 56.5 ± 11.24% in the cerebellum at one-month-old in the SD/Taurine group. The legends are common for all graphs. *P < 0.05 using multiple student's t-tests to compare the SD/Taurine group vs. SD controls.

A 2 g/L stock of taurine (Cat# T0625–100 G, Sigma-Aldrich; Saint Louis, MO, USA) was prepared, and then a concentration of 50 mg/L in drinking water was provided to one group of healthy pregnant Sprague Dawley rats at embryonic day 15 and continued throughout the lactation period until the offspring became one month old [6]. Taurine treatment was then restricted to male pups after one month of life and continued until eight months of age Fig. 1, Fig. 2, Fig. 3.

Once the male pups reached one month old, they were removed from their mothers and housed in groups of five rats per cage (acrylic; 34 cm × 44 cm × 20 cm). *Ad libitum* food (Formulab Diet 5008, containing 0.03% taurine; LabDiet; Saint Louis, MO, USA. Cat# 0,001,325) and filtered water or taurine solution were supplied, respectively. All experimental methods were carried out solely on the male pups under current Mexican regulation (NOM-062-ZOO-1999, SAGARPA), which is based on the Guide for the care and use of Laboratory Animals (2010). The rats were divided into two groups: (1) male Sprague Dawley rats that received no therapy (SD), and (2) male Sprague Dawley rats that received taurine supplementation (SD/Taurine). The groups were tested at different stages ranging from postnatal day 15 (PND15) to 8 months of age (8 M). As per statistical testing, all attempts were taken to lessen the number of individuals.

The mean ± SEM of at least three separate experiments is used, with a minimum *n* = 7 for motor tests done in triplicate. For parametric data in trials with two groups compared, a 2-tailed *t*-test was used. A one-way ANOVA and Dunnett's post hoc test were used for trials with more than two groups and one independent variable. *P < 0.05 when comparing treated rats to untreated controls using multiple student's t-tests. GraphPad Prism 6 (GraphPad Prism, San Diego, CA, USA. RRID: SCR 0,158,070) was used to perform all statistical analyses. The raw data may be found in the supplementary files.

### 2.2 Functional development

The primitive reflexes, also known as developmental reflexes, and the postural reactions are the first forms of automatic, stereotyped, and predictable movements that can help identify sensorimotor deficits [7]. With this in mind, we used the battery of motor tests given by [3] to analyze several distinctive neurological and neuromuscular markers in the development of both SD and SD/Taurine groups (Fig. 1).

The evaluation started at PND5 with the surface-righting reflex. The righting reflex is described as the “automatic reaction that enables a person to assume a normal standing position and maintain stability when changing positions” [8]. These righting reactions put the head and torso into mutual alignment with one another [7,8]. The righting reflex in rodents is the capacity of a pup to turn itself onto its pawns from a supine position (face up), and this reflex usually appears at PND5 [3]. PND5 pups were laid on their backs on a cotton sheet and kept in place by the evaluator for 5 s. After that, the pups were freed, and the time it took for the pup to resume the prone posture (face down) was recorded. The test was repeated three times for each pup, and the average value was used to determine the mean ± SEM of at least 7 pups for each group.

The next sensorimotor test was evaluated at PND9 when the pups’ eyes remain closed, and they perceive the outside world through the senses such as hearing. Hearing allows the subject to maintain balance thanks to many inner ear components linked to the cerebellum and vestibular nuclei in the brainstem through the vestibulocochlear nerve [9]; an impairment in the vestibular system may indicate direct injury to these nuclei. The cliff aversion test assesses vestibular imbalances [3] caused by vestibular system injury. Pups were positioned at the top of a closed wooden box (31×24×17 cm) for 30 s to explore the surroundings. The head and forelimbs were then put on the box cliff, and the time required to recede from the abyss was recorded. The test was repeated three times for each pup, and the average value was used to determine the mean ± SEM of at least 7 pups for each group.

We also evaluated a neuromuscular component through the gripping strength test and the limb suspension tests. The gripping strength test assesses the muscle strength of all four pawns simultaneously [3]. Pups were weighed on a digital rodent scale at PND11 and then placed on the center of a horizontal mesh (16×18 cm long × s1 mm^2^ thick) until all four limbs clutched the mesh. The evaluator then progressively rotated the mesh from a horizontal to a vertical position to resist gravity. The hanging impulse was calculated by measuring the falling time and angle (hanging impulse = body weight times the falling latency). The evaluator's average speed for manipulating the mesh should be a five-degree inclination per 2 s of testing. The test was repeated three times for each pup, and the average value was used to determine the mean ± SEM of at least 7 pups per group [3,5].

Finally, the strength in the forelimbs and hindlimbs was evaluated. PND13 pups were hanged by their forelimbs from a 0.5 mm thick stainless steel wire that crossed the top of a 3.5 L clear plastic container with a cushioned floor. The time for the pup to fall into the cushioned floor was considered the latency time. Next, the pups were suspended by their hindlimbs on the edge of a transparent glass container (15 cm tall × 6 cm wide) with smooth interior walls and a cushioned floor. The latency time was defined as the time it took for the puppy to fall onto the cushioned floor. The test was repeated three times for each pup, and the average value was used to calculate the mean ± SEM of at least 7 pups per group. It is essential to wait at least 30 min between front limbs and hindlimbs evaluation to avoid muscle fatigue in the subjects [3,10].

### 2.3 Nitric oxide determination

Once the subjects reached the specific analysis age, the brains were extracted, washed with 100 mL of ice-cold isotonic saline solution, and placed upside down on an ice-cold plate for dissection. The medulla oblongata, the brainstem, the cerebellum, the subcortical nuclei, and the cerebral cortex (*n* = 3 rats for each group) were sectioned, and the tissues were immediately deposited in 1.5 mL microcentrifuge tubes and kept at −80 °C until use. On the day of the experiment, the tissues were thawed at 4 °C. Once defrosted, the tissues were mechanically homogenized in 300 µL of phosphate-buffered solution (PBS) at pH 7.4. Afterward, the lysates were centrifuged at 12,500 rpm for 30 min at 4 °C (Centrifuge Z 216 MK, HERMLE Labortechnik; Wehingen, Germany), and the nitrites in the supernatants were quantified. Because nitric oxide (NO) can be a very volatile molecule, its presence in the tissue was determined indirectly by measuring the nitrite (NO_2_^−^) formation with the colorimetric Griess test. The chromogenic reaction was initiated in 100 µL of tissue supernatant by adding 100 µL of freshly prepared Griess reagent (0.1% N-(1-naphthyl) ethylenediamine dihydrochloride and 1.32% sulfanilamide in 60% acetic acid, 1:1 at 4 °C). Subsequently, the absorbance was measured at 540 nm with a SmartSpec 3000 spectrophotometer (Bio-Rad; Hercules, CA, USA) and interpolated from a NaNO_2_ standard curve (concentrations ranging from 1 to 10 µM) to determine the NO_2_^−^ concentration in the samples. To evaluate age-dependent variations, values were adjusted using the protein concentration per mg of tissue [2] and then normalized versus the median at PND15 [1] (Fig. 2).

### 2.4 Lipid peroxidation

On the same day as nitric oxide determination, malondialdehyde + 4-hydroxyalkenals (MDA + 4-HDA) levels were quantified in 200 µL of the same sample supernatant. The colorimetric reaction was generated by combining 200 µL of supernatant with 650 µL of 10.3 mM N-methyl-2phenyl-indole (Sigma-Aldrich; Saint Louis, MO, USA) diluted in acetonitrile:methanol (3:1) and 150 µL of methanesulfonic acid (Sigma-Aldrich; Saint Louis, MO, USA). This last reagent needs to be added to the reaction mixture as fast as possible using a glass pipette. Afterward, the three-component reaction mixture was vortexed, incubated in a water bath at 45 °C for 1 h, then centrifuged at 3000 rpm for 10 min. A Smart-Spec 3000 spectrophotometer (Bio-Rad; Hercules, CA, USA) was used to measure the absorbance of the reaction mixture's supernatant at 586 nm. To quantify the MDA + 4-HDA levels in the samples, absorbance data were interpolated from a standard curve in the concentration range of 0.5 to 5 µM of 1,1,3,3-tetramethoxypropane (10 mM stock). To detect the age-dependent changes, values were adjusted using the protein concentration per mg of tissue [2] and then normalized with the median at PND15 (Fig. 3).

## Ethics Statement

All experiments comply with the ARRIVE guidelines and were carried out under the U.K. Animals (Scientific Procedures) Act, 1986 and associated guidelines, EU Directive 2010/63/EU for animal experiments, or the National Institutes of Health guide for the care and use of laboratory animals (NIH Publications No. 8023, revised 1978).

## CRediT authorship contribution statement

**Viridiana Vargas-Castro:** Methodology, Formal analysis, Investigation, Writing – original draft, Writing – review & editing. **Ricardo Gomez-Diaz:** Methodology, Investigation. **Victor M. Blanco-Alvarez:** Validation, Investigation. **Constantino Tomas-Sanchez:** Validation, Investigation. **Alejandro Gonzalez-Vazquez:** Formal analysis, Investigation. **Ana Karina Aguilar-Peralta:** Formal analysis, Investigation. **Juan A. Gonzalez-Barrios:** Resources, Funding acquisition. **Daniel Martinez-Fong:** Writing – review & editing. **Jose R. Eguibar:** Resources. **Araceli Ugarte:** Resources. **Guadalupe Soto-Rodriguez:** . **Eduardo Brambila:** Formal analysis. **Lourdes Millán-Perez Peña:** Resources. **Bertha Alicia Leon-Chavez:** Conceptualization, Project administration, Visualization, Supervision, Funding acquisition, Writing – review & editing.

## Declaration of Competing Interest

The authors declare that they have no known competing financial interests or personal relationships, which have or could be perceived to have influenced the work reported in this article.
